# Risk factors for Group B Streptococcus colonisation and disease in Gambian women and their infants

**DOI:** 10.1016/j.jinf.2015.12.014

**Published:** 2016-03

**Authors:** K. Le Doare, S. Jarju, S. Darboe, F. Warburton, A. Gorringe, P.T. Heath, B. Kampmann

**Affiliations:** a Imperial College, Norfolk Place, London W2 1PG, UK; b MRC Unit, Atlantic Road, Fajara, Gambia; c Public Health England, 61 Colindale Avenue, London NW9 5EQ, UK; d Public Health England, Manor Farm Road, Porton Down SP4 0JG, UK; e St George's University of London, Cranmer Terrace, London SW17 0RE, UK

**Keywords:** Neonatal infection, Group B Streptococcus, Vaccines, Immunity

## Abstract

**Objectives:**

To determine risk factors for GBS colonisation in Gambian mothers and in their infants from birth to day 60–89 of age.

**Methods:**

Swabs and breastmilk from mothers/infant pairs were collected and cultured on selective agar. Negative samples were analysed for GBS DNA via real-time PCR. Positive isolates were serotyped using multiplex PCR and gel-agarose electrophoresis.

**Results:**

Seven hundred and fifty women/infant pairs were recruited. 253 women (33.7%) were GBS-colonised at delivery. The predominant serotypes were: V (55%), II (16%), III (10%), Ia (8%) and Ib (8%). 186 infants were colonised (24.8%) at birth, 181 (24.1%) at 6 days and 96 at day 60–89 (14%). Infants born before 34 weeks of gestation and to women with rectovaginal and breastmilk colonisation at delivery had increased odds of GBS colonisation at birth. Season of birth was associated with increased odds of persistent infant GBS colonisation (dry season vs. wet season AOR 2.9; 95% CI 1.6–5.2).

**Conclusion:**

GBS colonisation is common in Gambian women at delivery and in their infants to day 60−89 and is dominated by serotype V. In addition to maternal colonisation, breastmilk and season of birth are important risk factors for infant GBS colonisation.

## Highlights

• Increased risk of infant GBS colonization is associated with maternal rectovaginal and breastmilk colonization.
• GBS serotype V is a major colonizing serotype in the Gambia, thus this antigen should be included in candidate vaccines.
• Midwife delivery, number of antenatal visits and season of birth are additional risk factors in low-income settings.

## Introduction

Group B Streptococcus (GBS) is a leading cause of neonatal infection worldwide and transmission occurs mainly from mother to child during the peripartum period.^1^ In the African setting, information on GBS disease prevalence remains sparse.^2^ Under-identification and under-reporting of GBS cases and deaths appear likely, especially where it is difficult to access healthcare. Maternal colonisation is the leading risk factor for both early and late onset GBS disease,^1^ yet in resource-poor countries little is known about maternal colonisation rates. Little is also known about maternal or infant colonising serotypes in African countries but reports indicate that serotype (ST) V may be important as both a colonising and invasive disease serotype,^3^, ^4^ as it was in the USA in the 1990s.^5^

The current leading candidate for maternal vaccination is a trivalent capsular polysaccharide protein conjugate vaccine containing ST Ia, Ib and III which is based on data on invasive disease serotypes in the USA, UK^6^ and South Africa.^3^ Here, we report the results of a longitudinal prospective cohort study to investigate the prevalence of colonising and invasive disease serotypes of Gambian women and their infants from delivery to three months of age.

## Methods

### Study design and participants

We undertook a prospective longitudinal cohort study in two government health centres offering antenatal care to women in the Fajara area of costal Gambia, a low-income country with an annual birth rate of 43.1/1000 population, neonatal sepsis rate of 4.42/1000 live births^7^ and neonatal mortality rate of 28/1000 live births.^8^ The combined birth rate of these two health centres is approximately 12,500 births annually. The health centres were selected to be representative of the level of care usually available to Gambian women.

The eligibility criteria for maternal participation in the study included all pregnant women over the age of 18 years who had a negative HIV test and were deemed to be at low risk for pregnancy complications (no evidence of pre-eclampsia, cardiomyopathy, maternal gestational diabetes, placenta praevia, twin pregnancy). Women were invited to deliver at the health centre and offered a confirmatory HIV test prior to enrolment. Women found to be HIV positive were referred for specialist ongoing care. Mothers were excluded if they were not planning to breastfeed or were unable to remain in the Fajara area for the first three months postpartum. Healthy infants over 32 weeks of gestation assessed using the Ballard score and weighing over 2.5 kg were included. Infants were excluded if they had obvious congenital abnormalities or required resuscitation at the time of delivery requiring transfer to a neonatal unit. Mother and infant pairs were recruited to the study between 1st January 2014 and 31st December 2014 if both mother and infant met the inclusion criteria. All eligible women and infants were recruited until the pre-defined sample size was reached. Participants were followed up daily at home until day 6 and then asked to return to clinic when the infant 60−89 days old for final follow up visit and vaccinations. If an infant died during the study a verbal autopsy was carried out to assess the potential cause of death.

### Ethics statement

Field workers explained the purpose of the study to eligible participants in their local language (Mandinka, Wolof, Fula, Jolla, Mangago) and each participant signed an informed consent form, or in case of illiteracy, thumb-printed and the consent form was signed by an impartial witness. The study was approved by the joint Gambian Government/Medical Research Council Research Ethics Committee, SCC 1350 V4.

### Procedures

#### Sampling

Rectovaginal swabs were taken from enrolled women presenting in labour to one of the health centres and cord blood was taken after delivery but prior to separation of the placenta. A screening questionnaire was completed after four hours postpartum and the infant checked for any abnormalities requiring medical intervention. The questions included ethnic origin, gravida, parity, maternal weight, blood pressure, haemoglobin concentration, use of medication/traditional medicines/antibiotics and vaccination in pregnancy, any illnesses in pregnancy, number of antenatal attendances, HIV status, education, diet, compound location and presence of cattle at the compound. Nasopharyngeal and rectal swabs were taken from all eligible infants at four hours after birth. Mothers were provided with soap and asked to wash their hands and wipe their breasts with alcohol wipes before hand expressing colostrum/milk within the first 12 h after birth, at day 6 and between days 60 and 89. Nasopharyngeal and rectal swabs were also taken from infants at day 6 of life and again at 60–89 days of life. Infants who were unwell before day 6 were assessed at home and referred for treatment as necessary. All sick infants had a blood culture taken on admission to hospital. At each visit a standardized questionnaire was completed in the local language documenting infant anthropometry, feeding, vaccinations, signs and symptoms of infant illnesses, use of antibiotic/traditional medicine and vital signs.

#### Laboratory procedures

Copan (for rectal and rectovaginal samples) and Dacron (for nasopharyngeal samples) swabs were collected in skim-milk tryptone glucose glycerol (STGG) transport medium, stored at 4 °C and transported to the Medical Research Council laboratories, The Gambia within 4 h of collection. On arrival the samples were vortexed briefly and immediately frozen at −70 °C until processing.

All swab specimens were inoculated into Todd–Hewitt broth supplemented with colistin and nalidixic acid and were processed for isolation of GBS using standard laboratory procedures.^1^ Presumptive positive GBS samples were identified by latex agglutination (Oxoid). Five colonies from positive samples were harvested into phosphate buffered saline (PBS) and subjected to real-time polymerase chain reaction (PCR).^9^ Negative samples were also subjected to confirmation by real-time PCR. All GBS positive isolates were then serotyped using conventional PCR and identified using gel PCR and agarose electrophoresis.^10^

#### Outcomes

The primary outcome was prevalence of ST-specific GBS colonisation in mothers and infants at birth, six days and between days 60–89 using microbiological culture and molecular techniques. Secondary outcomes were detection of GBS in breastmilk; infant acquisition and loss of GBS colonisation during the study follow up period and infant GBS disease (sample obtained from sterile site), as ascertained by positive microbiological culture and confirmatory PCR. Swabs were considered negative if no GBS was evident by culture and PCR and positive if GBS was found on culture and PCR. If swabs were negative on culture but positive on PCR, conventional PCR was performed to determine serotype. If the second PCR resulted in the identification of a GBS serotype the samples were deemed positive. If no serotype was identified or the DNA did not amplify, samples were deemed negative.

#### Statistical analysis

Calculated on the basis of the previously observed 24% colonisation rate,^11^ the intended sample size was 750 mothers, to provide at least 180 colonised women for serotype analysis (95% confidence interval (CI) 150–202 women) and 90 colonised infants (95% CI 72–107 infants). The sample size of 180 colonised women was chosen to ensure at least 10 samples of the least prevalent ST based on historical data from The Gambia (ST III (6%)),^11^ in order to allow longitudinal colonisation analysis.

Statistical analyses were completed using STATA version 12 (StataCorp 2013, Texas) and GraphPad Prism version 6.0 (GraphPad Software Inc, La Jolla, California). Descriptive statistics included the prevalence of colonisation at individual time points expressed as a proportion of the total number of participants. Odds ratios and 95% confidence intervals (CI) were calculated to determine risk of maternal and infant colonisation at birth in a single variable analysis. Adjusted odds ratios were then calculated using any variables from the single variable analysis with a p-value <0.2 using a backwards-stepwise procedure.

Analyses of the changes in infant colonisation over time were undertaken using longitudinal logistical regression. Adjusted odds ratios were then calculated using any variables from the single variable analysis with a p-value <0.2 using a backwards-stepwise procedure. New acquisition of GBS was defined as detection of a new serotype by culture or PCR that was not previously present. Clearance of colonisation was defined as a negative GBS culture or PCR for a specific serotype following a positive sample at the previous visit for the same ST. The log-rank test was used to examine differences in duration of colonisation between serotypes. Using an expected vertical transmission rate of 50%^1^ we calculated observed vs. expected statistics for risk of infant colonisation by ST. For comparison between our study and the study conducted in 1994, we calculated 95% confidence intervals for both studies. P-values <0.05 were considered significant.

#### Role of the funding source

The funders had no role in study design, data collection, data analysis, data interpretation, or writing of the report. All authors had full access to all the data and the corresponding author had final responsibility for the decision to submit for publication.

## Results

### Demographics

Between 15th January 2014 and 31st January 2015 we recruited a total of 750 mothers and their infants to the study. All mothers met the inclusion criteria except one, who was admitted to hospital on day three post-partum severely unwell and subsequently tested positive for HIV1, despite a negative test at 20 weeks of gestation. She and her infant were referred for further management and samples excluded from further analysis. Of the remaining eligible women and infants, 722 completed the day 6 visit (median age at follow up 8 days, IQR 7–9) and 684 completed 60–89 days follow up (median age at follow up 62 days, IQR 60–63). The main reasons for not completing follow up were: 31 women moved out of area, 20 women whose partners were not in agreement with continued study participation, one maternal HIV, nine infant deaths, five declined for other reasons. Participant flow diagram and demographic characteristics of mother–infant pairs are outlined in Supplementary Fig. 1.

### Antibiotic treatment

Over the course of the study, 77 women received antimicrobials during pregnancy: 34 for urinary tract infection; 10 for pneumonia; 14 for vaginal discharge; 9 for malaria and 10 for non-malarial fever. Of these, 73 women received amoxicillin (median time prior to delivery 42 days [IQR 18–63 days]); 16 received antimalarial therapy (median 38 [31–41]) and three received erythromycin. In the three months of follow up, 51 infants required antibiotic therapy: 34 received ampicillin and gentamicin for presumed neonatal sepsis (median 18 days old [IQR 4–34]); 10 received amoxicillin or cloxacillin for skin infections (20 days [10–58]); 7 received other antibiotics (18 days [11–23]). Antibiotic therapy was included in adjusted analyses and had no impact on colonisation of mother or infant at any time point.

### Prevalence and risk factors for maternal colonisation

The overall prevalence of recto-vaginal GBS colonisation at delivery was 33.7% (253 women). In single variable analysis maternal weight >50 kg, maternal haemoglobin <10 g/dL, more than one previous stillbirth, more than one antenatal clinic visit, midwife delivery, giving birth during the dry hot or wet seasons, birth weight over 3 kg and gestation under 34 weeks were associated with increased risk of GBS colonisation (Table 1). In adjusted analysis, maternal haemoglobin <10 g/dL, history of more than one previous stillbirth, attendance at delivery by a midwife rather than a trained birth attendant and giving birth during the wet or dry hot season were associated with increased odds of maternal GBS colonisation at delivery (Table 2).

### GBS colonisation dynamics at birth

**Figure 1:**
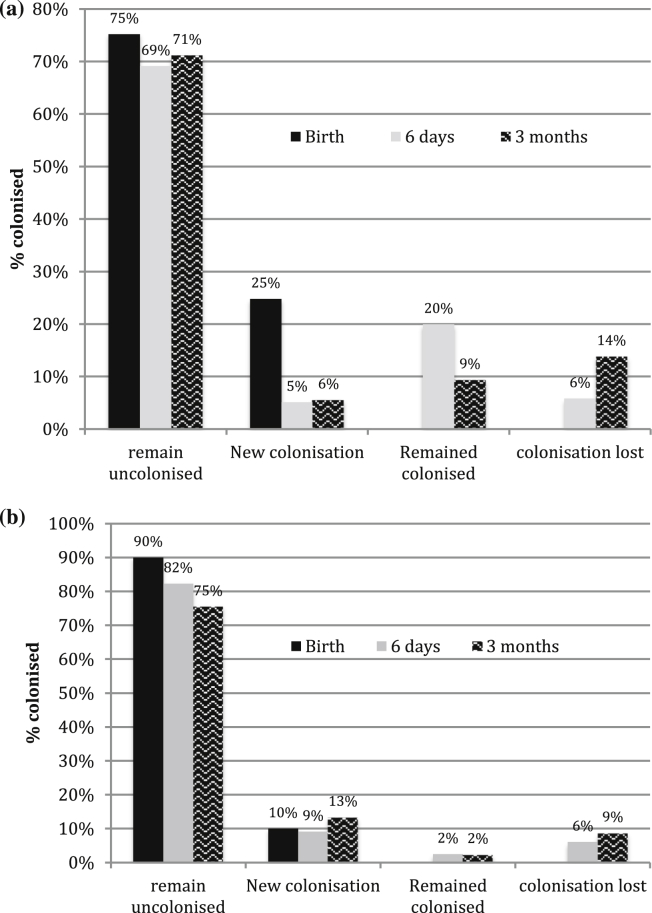
a – infant GBS colonisation status over time. Bars represent percentage of infants colonised at each of the time points; black bar = birth, grey = day 6 and patterned = three months. b – breastmilk colonisation over time. Bars represent percentage of infants colonised at each of the time points; black bar = birth, grey = day 6 and patterned = three months.

There were 253 colonised mothers and 186 colonised infants at birth, of whom 146 were born to colonised women (57.7%). Table 3 describes the number of colonised infants at birth, day 6 and at 60−89 days of age according to colonisation site. Fig. 1a and b demonstrate colonisation dynamics at each time point.

Compared to mother/infant pairs who were not colonised at birth, mother/infant pairs were more likely to both be colonised if mother's weight was below 50 kg, infant was born at less than 37 weeks of gestation, born during the dry hot or wet seasons, mothers with more than one previous stillbirth, mothers attending antenatal clinic more than once, infants delivered by midwives, infants born with a birth weight below 3.3 kg or receiving breastmilk colonised with GBS (Table 4).

In adjusted analysis, compared to mother/infant pairs who were not colonised at birth, mother/infant pairs were more likely to both be colonised if birth occurred in the dry hot or wet seasons, mothers with one or more previous stillbirths or spontaneous abortions, infants born before 34 weeks of gestation. Compared to mother/infant pairs where neither was colonised, mother/infant pairs where the mother was colonised but not the infant were more likely if the mother had more than one previous stillbirth, or if the mother gave birth during the wet season. Infant colonisation in the absence of maternal colonisation (n = 40) was associated with gestation before 34 weeks (Table 5).

Most notably, compared to mother/infant pairs at birth where neither were colonised, infants born to mothers who had GBS cultured in their breastmilk were more likely to be colonised with GBS (Table 5).

### GBS colonisation dynamics at birth, day 6–9 and day 60–89

Infants born to colonised mothers had an increased risk of colonisation at birth and day 6–9 if born during the dry hot season or if born before 34 weeks of gestation. For all variables the risk of colonisation decreased over time (Table 6).

Infants born to non-colonised mothers had an increased risk of being GBS colonised on day 6–9 and at day 60–89 if born during the wet season. At birth infants were more likely to be colonised if breastmilk was colonised and they remained more likely to be colonised at both 6–9 days and day 60–89.

### Serotype distribution

**Figure 2:**
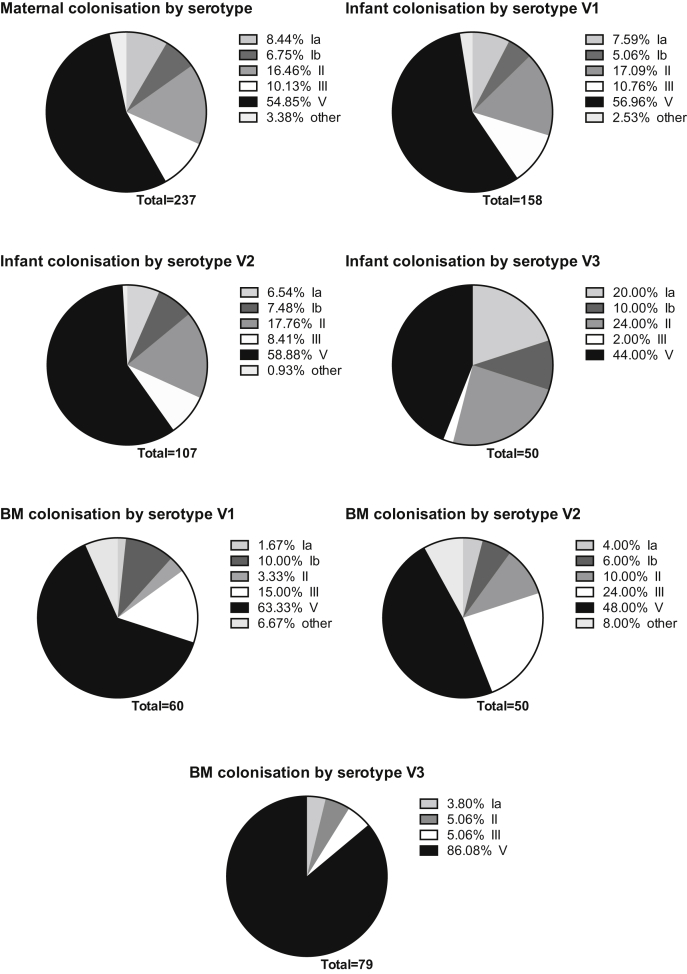
Colonising serotype distribution by isolation site and time point. Maternal rectovaginal colonisation at delivery; breastmilk and infant colonisation by time point; BM = breastmilk; Ia = serotype Ia, Ib = serotype Ib, II = serotype II, III = serotype III, V = serotype V.

The predominant maternal rectovaginal colonising serotype was ST V in 54.9% of colonised mothers. Mothers' breastmilk was more likely to be colonised with ST V (p < 0.001) at all time points than any other serotype. Infants were more likely to be colonised with ST V at birth (p = 0.004), day 6–9 (p = 0.004) and day 60–89 (p = 0.009) than any other serotypes. At birth, infants were more likely to be colonised with ST III and V if their mothers were colonised with ST III and V (x^2^ = 24.4, p < 0.001) compared to mothers colonised with other serotypes. Fig. 2 outlines the percentage of different serotypes at different time points in infants and in breastmilk.

### Neonatal sepsis

During the study period, 51 infants (6.8%) presented with signs of infection (68 per 1000 live births): 18 with signs of pneumonia, 22 with signs of other febrile illness, ten with skin infections and one with signs of meningitis. One child presenting on day 6 of life with irritability and poor feeding, had a positive CSF culture for GBS and was treated for 21 days with ampicillin and gentamicin (invasive GBS disease incidence: 1.3 per 1000 live births). This infant was born to a mother who had ST V cultured from both rectovaginal swab and breastmilk at birth and day 6 of life and the infant swab was also positive at birth and day 6 for ST V. The CSF sample was not typed due to laboratory constraints.

Nine infants died during the study period. Based on verbal autopsy, six deaths were due to pneumonia (median age at death 7 days [IQR 2–35]); three due to sepsis (median age 6 days [1–48]). Blood cultures were negative in all cases. One infant was born to a colonised mother and was also colonised with GBS at the time of birth but was not colonised on day 6. This infant died of presumed sepsis on day 48 of life. No other infants who died or their mothers were colonised with GBS at time of birth or day 6 of life. All infants died prior to the final follow-up appointment. It is therefore, not possible to determine whether any of these deaths were due to GBS disease.

## Discussion

In this first colonisation study of Gambian mothers and their infants for 25 years and the first longitudinal study undertaken in this population, we demonstrate high prevalence of GBS colonisation in mothers at birth and in their infants up to 90 days of life. In addition, we demonstrate that infants were more likely to be colonised if GBS was isolated from their mother's breastmilk. The overall prevalence of maternal GBS in our population is higher than in the original study by Suara et al. (1992) (Suara 22% colonised (95% CI 15.04–28.96) vs. present study 33.7% colonised (29.63–36.37); p = 0.007).^11^ However, the previous study likely under-identified GBS colonisation as selective medium was not used. An additional feature that may account for a lower colonisation rate was the collection of vaginal but not rectal swabs, which are known to have a higher yield for GBS.^12^ The prevalence of GBS colonisation in our study is similar to the reported pooled prevalence in South African women (30.4%).^3^

Our finding that mothers with more than one stillbirth demonstrated an increased odds of having a colonised infant are in concordance with Monari et al., who identified stillbirth as a risk factor for invasive EOGBS disease.^13^ This phenomenon has also been noted in non-human primates,^14^ but there are currently little human data on the association between stillbirth, GBS colonisation and invasive disease. This finding could be important in reducing adverse pregnancy outcomes especially following introduction of any new vaccine.

The prevalence of infant colonisation in our study is similar to previous studies in Europe^15^, ^16^, ^17^, ^18^ but higher than those from other most African countries,^19^ probably due to the differences in microbiological methods. To our knowledge this is the first longitudinal study of infant colonisation and demonstrates that GBS colonisation rates remain high up to three months of life. We demonstrate that the overwhelming risk factor for infant colonisation at all time points was having a mother who was GBS colonised at the time of birth. We also established that delivery before 34 weeks of gestation increased the odds of GBS colonisation at birth. Similar studies have indicated that risk factors for infant colonisation include maternal rectovaginal colonisation, preterm delivery, low birth weight and maternal urinary tract infection.^1^, ^15^, ^18^, ^20^ However, unlike these studies we did not identify low birth weight as a risk factor, probably because we only recruited infants weighing more than 2.5 kg.

Several case reports suggest that isolation of GBS in breastmilk is implicated in late onset GBS disease.^21^, ^22^ Our data suggest that GBS colonisation in breastmilk is a risk factor for neonatal colonisation, both at delivery and for persistence of infant colonisation throughout the first three months of life. This is an important finding in a population such as this with high rates of breast-feeding. The mechanism for this is not clear; it is possible that GBS enters the mammary glands following suckling from a colonised infant, although there is also evidence that normal gut microbiota may pass into the breast via the entero-mammary circulation.^23^ Whether it is also a risk factor for late onset disease in this population is not known. Conversely, it might conceivably be a protective factor through induction of immunity.^24^ The relevance of this finding requires further study.

It is interesting to note that risk factors for maternal colonisation include midwife delivery and increasing number of antenatal visits. This phenomenon has not been previously noted in resource-poor settings where hygienic birth practices may be compromised. GBS has been implicated in nosocomial outbreaks in several hospitals globally,^25^, ^26^ although the number of reported outbreaks in resource-rich countries has greatly reduced since the implementation of GBS screening programmes. It is conceivable that unclean birth practices, especially in times of clean water shortage, such as the dry hot season or during the wet season may facilitate the fecal-oral spread of GBS between colonised and non-colonised mothers and their infants.

A large proportion of women were colonised with ST V, similar to the previous smaller study by Suara et al., which identified 32 colonised pregnant women of whom 12 (30%) were colonised with ST V.^11^ ST V has been identified as an important neonatal and adult colonising and disease causing serotype globally.^3^, ^27^, ^28^ Additionally, in our cohort, ST V was the predominant serotype cultured in breastmilk and ST Ib and V were associated with longer median colonisation duration compared to other serotypes. A study of pregnant women from South Africa found a high prevalence of GBS colonisation throughout pregnancy, with ST V and ST III colonisation lasting longer than other serotypes (ST V mean duration 8.6 weeks; 95% CI 6.8–10.4 vs. ST III 9.2 weeks; 95% CI 8.1–10.2).^3^ The difference in serotype distribution and duration of colonisation between these studies may be due to population differences between South Africa and the Gambia. There are no other longitudinal infant colonisation studies with which to compare our results. Our results may also indicate differences in maternally derived antibody that might prevent colonisation of certain serotypes in these diverse populations. In the most recent study from South Africa, the authors demonstrate that it is the acquisition of a new serotype that is associated with EOS rather than the persistent serotype, probably due to the absence of maternal antibody to this new serotype.^29^

Our study has several limitations. First, our results are limited by the sensitivity of detection of GBS on selective media, estimated at 85%^30^ and by the fact that we identified the serotype by conventional PCR. This may imply that we have underestimated low levels of colonisation in infants where DNA was below the limit of detection. Second, we identified only one culture-confirmed case of GBS invasive disease. The low number of positive invasive isolates may represent an underestimation of the burden of GBS disease in this population. Several infants died prior to recruitment or after recruitment but of early onset pneumonia without a culture-positive diagnosis and it is conceivable that they may have succumbed to early onset GBS disease. There are no other data from the Gambia regarding the burden of GBS disease. Although we recognize the limitations of its approach, the only other evidence for GBS disease in the Gambia is shown by a retrospective review of positive cultures from the MRC hospital over 8 years that identified 17 positive cultures from infants under 90 days of age which is consistent with our disease estimate of 1.3/1000 live births.^31^ Finally, sampling at 4 h post-birth may indicate contamination with maternal secretions rather than established colonisation at birth. However, the fact that few infants lost colonisation at day 6 indicates that colonisation was persistent over the first week of life.

GBS capsular polysaccharide-protein conjugate vaccines are being developed, making the findings of our study important to ensure that appropriate serotypes are included in candidates that would be relevant to all African populations. Information about GBS colonisation and factors affecting acquisition and duration of colonisation will be helpful in considering strategies for reducing the burden of infant GBS disease worldwide.

## Author contribution

KLD conceived the original idea, designed and performed the laboratory and data analysis and drafted the manuscript. SJ and SD performed and interpreted the laboratory analysis and contributed to the manuscript preparation. AG, PH and BK developed the original research idea and contributed to the manuscript preparation. FW advised on the statistical analysis and commented on drafts of the manuscript.

## Funding

This work was supported by a Wellcome Trust Clinical Research Fellowship to KLD (WT104482MA) and the Thrasher Research Fund (BK: 12250). BK is also supported by grants from the UK MRC (MC_UP_A900/1122, MC_UP_A900/115) and the UK Medical Research Council (MRC) and the Department for International Development (DFID) under the MRC/DFID Concordat arrangement.

## Conflict of interests

KLD, SJ, SD, AG declare no conflict of interests. PTH is an advisor to Novartis and Pfizer regarding GBS vaccines. BK is an advisor for Pfizer regarding GBS vaccines.

## Figures and Tables

**Table 1:** Mother and infant demographics and odds ratios for maternal GBS colonisation at delivery.

		Total	Colonised	Non-colonised	p-value	OR (95% CI)
**Maternal age yrs**	<20	110	40	70	0.78	1
20–24	262	81	181		0.78 (0.5–1.3)
*Mean: 25 (IQR 18–41)*	25–29	203	71	132		0.94 (0.6–1.5)
30+	175	61	114		0.99 (0.6–1.7)
**Ethnicity**	Mandinka	315	115	200	0.38	1
Wolof	108	36	72		0.9 (0.5–1.4)
Fula	126	45	81		1 (0.6–1.5)
Jola	118	33	85		0.7 (0.4–1.0)
Other	81	23	58		0.7 (0.4–1.2)
Missing	2	1	1		
**Maternal weight (kg)**	<50	27	7	20	0.03*	1
50–70	424	128	296		1.2 (0.5–3.0)
70–90	148	61	87		2.0 (0.8–5.0)
>90	145	56	91		1.8 (0.7–4.4)
Missing	6	3	3		
**Maternal illness in pregnancy**	Yes	128	45	83	0.32	1
No	504	165	339		1.1 (0.7–1.7)
Missing	118	43	75		
**Maternal Hb below 10 g/dL**	No	99	42	57	0.04*	1
Yes	651	211	440		0.5 (0.3–0.9)
**Maternal antibiotics in pregnancy**	No	505	166	339	0.74	1
Yes	128	44	84		1.1 (0.7–1.6)
Missing	117	74	43		
**Gravida**	1	208	67	141	0.44	1
*Median: 2 (IQR 1–4)*	2–4	370	121	249		1 (0.7–1.5)
5+	169	63	116		1.3 (0.8–2.0)
Missing	3	1	2		
**Parity**	0–1	212	68	144	0.59	1
*Median: 1 (IQR 0–4)*	2–4	468	164	304		1.1 (0.8–1.6)
5+	67	20	47		1.1 (0.7–1.7)
Missing	3	1	2
**Living children**	0	230	80	150	0.17	1
*Median: 1 (IQR 0–3)*	1–4	465	155	311		0.9 (0.7–1.3)
5+	53	17	36		0.9 (0.5–1.7)
Missing	2	2	0		
**Spontaneous abortion**	0	649	213	436	0.09	1
*Median 1(IQR 0–2)*	1	53	20	33		1.2 (0.7–2.2)
2+	14	7	7		1.5 (0.8–2.7)
Missing	34	16	18		
**Stillbirths**	0	641	207	434	0.05**	1
1	75	29	46		0.9 (0.4–2.1)
2+	28	15	13		3.6 (1.2–10.5)
Missing	6	2	4		
**Number of ANC visits**	1	303	85	218	0.01*	1
2+	447	168	279		1.5 (1.1–2.1)
**Attendance by midwife or TBA**	Midwife	436	168	268	0.001*	1
TBA	254	67	187		0.5 (0.4–0.8)
Missing	60	37	23		
**Birth season**	Dry cool	280	55	225	0.000*	1
Dry hot	175	85	90		3.9 (2.5–5.9)
Wet	295	113	18		2.5 (1.7–3.7)
**Infant sex**	Male	369	122	247	0.59	1.1 (0.8–1.5)
Female	381	131	250		
**Birth weight (kg)**	2.5–3	260	73	187	0.04*	1
3.1–4	464	169	295		1.5 (1.1–2.0)
>4.0	26	11	15		1.9 (0.8–4.3)
**Gest. age at birth (wks.)**	<34	56	18	38	0.03*	1
34–37	231	83	148		0.7 (0.4–1.1)
38+	463	152	311		1.1 (0.7–1.7)

IQR = inter-quartile range; ANC = antenatal clinic; TBA = trained birth attendant. * p-values <0.05 were considered significant. ** p-value = 0.0475.

**Table 2:** Multivariable analysis: maternal GBS colonisation at time of delivery.

		AOR (95% CI)	p-value
Maternal weight (kg)	<50	1	
50–70	1.0 (0.4–1.6)	0.96
70–90	1.7 (0.6–4.5)	0.30
>90	1.4 (0.3–4.7)	0.68
Number of ANC visits	1	1	
2+	1.3 (0.9–1.8)	0.16
Maternal Hb below 10 g/dL	No	1	
Yes	0.7 (0.4–0.9)	0.04*
Attendance by midwife or TBA	Midwife	1	
TBA	0.6 (0.4–0.9)	0.03*
Previous stillbirths	0	1	
1	0.9 (0.4–2.0)	0.86
2+	5.4 (1.6–18.0)	0.009*
Birth season	Dry cool	1	
Dry hot	2.0 (1.3–3.1)	0.004*
Wet	1.8 (1.2–2.7)	0.008*

ANC = antenatal clinic, Hb = haemoglobin; TBA = trained birth attendant. * p-values <0.05 were considered significant.

**Table 3:** Dynamics of colonisation by site and time point.

*Birth n = 750; 253 colonised mothers (% total)*	
Mother and infant colonised	146 (19.5% total; 57.7% born to colonised mothers)
Infants colonised, mothers not colonised	40 (5.3)
Total colonised infants	186 (24.8)
Mothers colonised, infants not colonised	107 (14.3)
Neither mother nor infant colonised	457 (60.9)
*Site of colonisation at birth (% colonised infants)*	
Nasopharyngeal and rectal	96 (51.6)
Rectal only	68. (36.6)
Nasopharyngeal only	22 (11.8)
*Site of colonisation day 6–9; n = 722 (% of the 181 colonised infants)*	
Infant rectal colonisation only	95 (52.5)
Infant nasopharyngeal colonisation only	29 (16.0)
Infant nasopharyngeal and rectal	57 (31.5)
*Site of colonisation day 60–89; n = 684 (% the 94 colonised infants)*	
Infant rectal colonisation only	57 (60.6)
Infant nasopharyngeal colonisation only	26 (27.7)
Infant nasopharyngeal and rectal	11 (11.7)

**Table 4:** Characteristics of mother infant pairs according to colonisation status at birth.

	Total	M+I+	M+I−	M−I+	M−I−	p-value
**Maternal age**						0.96
<20	110	23	17	2	68	
20–24	262	43	38	19	162	
25–29	203	46	25	11	121	
30+	175	34	27	8	106	
**Ethnicity**						0.10
Mandinka	315	63	52	23	177	
Wolof	108	23	13	8	64	
Fula	126	28	17	2	79	
Jola	118	21	12	3	82	
Other	81	11	12	4	54	
Missing	2	0	1	0	1	
**Maternal weight (kg)**						0.04*
<50	27	2	5	4	16	
50–70	424	74	54	21	275	
70–90	148	36	25	3	84	
90+	145	32	23	9	81	
Missing	6	3	0	3	0	
**Mother ill**						0.48
Yes	128	24	21	3	80	
No	504	97	68	25	314	
Missing	118	25	18	12	63	
**Maternal Hb <10 g/dL**						0.11
No	99	24	18	2	55	
Yes	651	122	89	33	403	
**Maternal antibiotics in pregnancy**						
No	505	99	92	0	314	0.91
Yes	128	23	24	0	81	
**Gravida**						0.44
1	208	39	28	13	128	
2–4	370	67	54	17	232	
5+	169	39	25	10	95	
Missing	3	1	0	0	2	
**Parity**						0.94
0–1	212	39	29	13	131	
2–4	468	96	68	24	280	
5+	67	10	10	3	44	
Missing	3	1	0	0	2	
**Living children**						0.66
0	230	43	37	14	136	
1–4	465	94	61	25	285	
5+	53	7	9	1	36	
Missing	2	2	0	0	0	
**Spontaneous abortion**						0.09
0	649	121	92	31	405	
1	53	14	6	4	29	
2+	14	7	1	0	6	
Missing	34	11	0	5	18	
**Stillbirths**						0.03*
0	641	119	87	32	403	
1	75	20	9	6	40	
2+	28	5	10	2	11	
Missing	6	2	1	0	3	
**Number of ANC visits**						0.002*
1	303	58	27	15	203	
2+	447	88	80	25	254	
**Midwife delivery**	436	108	60	20	248	<0.001*
TBA	254	29	38	12	175	
Missing	60	9	9	8	34	
**Infant sex**						0.71
Male	369	72	50	23	224	
Female	381	74	57	17	233	
**Season**						<0.001*
Dry cool	280	22	33	17	208	
Dry hot	175	66	19	3	87	
Wet	295	58	55	20	162	
**Birth weight (kg)**						0.02*
2.5–3	260	44	29	20	167	
3.1–4	464	95	74	18	277	
>4.0	26	7	4	2	13	
**Gestation (weeks)**						0.04*
<34	56	13	5	4	34	
34–37	231	57	26	12	136	
38+	463	76	76	24	287	
**Breastmilk colonised**						<0.001*
Yes	64	31	18	7	8	
No	600	101	77	31	391	
Missing	86	14	12	2	58	

M+I+ = mother and infant colonised; M+I− = mother colonised, infant not colonised; M−I+ = mother not colonised, infant colonised, M−I− = neither mother nor infant colonised; TBA = trained birth attendant; ANC = antenatal clinic.

**Table 5:** Multivariable analysis of maternal/infant pairs by GBS colonisation status at time of delivery.^a^

	M+I+ OR (95% CI)	M+I− OR (95% CI)	M−I+ OR (95% CI)
**Season**			
Dry cool	1	1	1
Dry hot	8.3 (3.9–17.6)	1.8 (0.8–3.9)	0.4 (0.1–1.4)
Wet	3.5 (1.8–6.8)	2.3 (1.3–4.3)	1.1 (0.5–2.5)
**Breastmilk colonised**			
Yes	21.0 (8.6–51.3)	11.5 (4.5–29.4)	7.5 (2.0–27.5)
No	1	1	1
**Gestation (weeks)**			
<34	1	1	1
34–37	0.9 (0.9–1.0)	1.0 (0.9–1.1)	0.9 (0.8–1.0)
38+	0.4 (0.2–1.0)	0.5 (0.3–1.6)	0.9 (0.8–1.0)
Midwife delivery	1	1	1
TBA	0.9 (0.5–1.6)	1.0 (0.6–1.8)	0.6 (0.2–1.3)
**Number of antenatal clinic visits**			
1	1	1	1
2+	1.1 (0.7–1.9)	1.2 (0.8–2.1)	1.3 (0.6–2.8)
**Stillbirths**			
0	1	1	1
1	2.5 (1.0–8.7)	2.0 (1.0–4.7)	1.4 (0.3–5.9)
2+	2.4 (0.9–4.1)	2.2 (0.9–2.1)	ND
**Spontaneous abortion**			
0	1	1	1
1	0.9 (0.5–1.4)	0.8 (0.3–2.1)	1.0 (0.2–5.5)
2+	1.2 (1.1–2.3)	0.3 (0.0–2.2)	2.0 ND
**Gravida**			
1	1	1	1
2–4	1.0 (0.9–1.0)	1.1 (0.6–2.0)	1.0 (0.8–1.2)
5+	1.0 (0.5–2.0)	0.8 (0.4–1.8)	0.8 (0.3–2.3)

M+I+ = mother and infant colonised; M+I− = mother colonised, infant not colonised; M−I+ = mother not colonised, infant colonised, M−I− = neither mother nor infant colonised. 1 = adjusted odds ratios are compared to this group; TBA = trained birth attendant.

a M−I− = base group.

**Table 6:** Single variable analysis of infant GBS colonisation dynamics over time compared to birth.

		Birth OR (95% CI)	Day 6	Day 60–89
		OR (95% CI)	OR (95% CI)
**Mother GBS colonised at delivery**				
Birth season	Dry cool	1	0.2 (0.1–0.6)	0.1 (0.0–0.4)
Dry hot	4.6 (2.2–9.7)	1.9 (1.0–4.1)	0.6 (0.2–1.3)
Wet	1.4 (0.7–2.8)	0.7 (0.3–1.5)	0.3 (0.1–0.7)
Living children	0	1	0.5 (0.3–0.9)	0.2 (0.1–0.4)
1–4	0.9 (0.5–1.6)	0.4 (0.2–0.7)	0.1 (0.0–0.3)
5+	0.9 (0.3–2.6)	0.6 (0.2–1.8)	0.2 (0.0–0.9)
Gestation at birth (wks.)	<34	1	0.4 (0.1–1.4)	ND
34–37	0.9 (0.3–2.4)	0.3 (0.1–0.9)	0.2 (0.1–0.7)
38+	0.4 (0.1–1.0)	0.2 (0.1–0.5)	0.0 (0.0–0.1)
Infant weight (kg)	2.5–3	1	0.5 (0.2–1.0)	0.1 (0.0–0.3)
3.1–4.0	1.2 (0.4–3.8)	0.7 (0.3–0.9)	0.1 (0.1–0.3)
>4.0	0.6 (0.1–2.3)	0.3 (0.1–0.7)	0.1 (0.0–0.6)
BM colonised	Yes	0.9 (0.5–1.7)	0.4 (0.2–0.8)	0.3 (0.1–0.7)
No	1	0.4 (0.3–0.6)	0.1 (0.1–0.2)
Infant sex	Male	1	0.4 (0.3–0.7)	0.1 (0.1–0.3)
	Female	0.7 (0.4–1.2)	0.4 (0.2–0.6)	0.1 (0.1–0.3)
**Mother not GBS colonised at delivery**				
Birth season	Dry cool	1	1.4 (0.5–4.3)	0.8 (0.2–2.8)
Dry hot	1	1.3 (0.3–5.3)	4.7 (1.7–13.5)
Wet	2.1 (0.8–6.0)	4.1 (1.6–10.5)	0.2 (0.0–1.9)
Living children	0	1	1.8 (0.5–5.7)	1.7 (0.5–5.6)
1–4	1.0 (0.3–2.9)	2.2 (0.8–5.9)	0.7 (0.2–2.3)
5+	2.0 (0.1–7.1)	1	0.8 (0.1–7.3)
Gestation at birth (wks.)	<34	1	3.3 (0.3–34.4)	1
34–37	1.6 (0.2–13.4)	2.5 (0.3–20.6)	1.4 (0.2–13.0)
38+	1.1 (0.1–9.0)	2.2 (0.3–17.3)	1.3 (0.2–10.8)
Infant weight (kg)	2.5–3.0	1	1.2 (0.4–3.8)	0.6 (0.1–2.3)
3.1–4.0	0.5 (0.2–1.4)	1.2 (0.5–2.9)	0.6 (0.2–1.7)
>4.0	0.9 (0.2–2.3)	1.7 (0.9–4.9)	0.4 (0.3–2.2)
BM colonised	Yes	14.2 (4.0–50.8)	6.9 (2.5–19.1)	3.3 (1.2–11.0)
No	1	2.0 (0.9–4.2)	1.0 (0.4–2.5)
Infant sex	Male	1	1.4 (0.6–3.1)	0.8 (0.3–2.0)
	Female	0.3 (0.1–1.0)	1.1 (0.5–2.5)	0.5 (0.2–1.5)

## Appendix A Supplementary data

Supplementary Figure 1 Flow diagram to demonstrate recruitment, retention and loss to follow up. The recruitment process at each stage of the field study with reasons for exclusion or loss to follow up. Loss to follow-up was 3.6% at end of day 6 and 8.8% by the end of day 60–89 of life.
