# Supplementary material for: Risk factors for Group B Streptococcus colonisation and disease in Gambian women and their infants
Source: J Infect. 2016 Mar;72(3):283–94. doi: 10.1016/j.jinf.2015.12.014 (PMC4769314; doi:10.1016/j.jinf.2015.12.014)
Supplement: Supplementary file 1 — Supplementary Figure 1 Flow diagram to demonstrate recruitment, retention and loss to follow up. The recruitment process at each stage of the field study with reasons for exclusion or loss to follow up. Loss to follow-up was 3.6% at end of day 6 and 8.8% by the end of day 60–89 of life. [file mmc1.docx]

**Supplementary Figure 1 – Flow diagram to demonstrate recruitment, retention and loss to follow up**
